# Effective Treatment of Cornu Cutaneum With Pachaieruvai: A Traditional Siddha Medicine

**DOI:** 10.7759/cureus.81326

**Published:** 2025-03-28

**Authors:** Saravanasingh Karan Chand Mohan Singh, Suresh K, A Jayakalaiarasi, Arun Prakash, Renga Sundari

**Affiliations:** 1 Department of Maruthuvam, National Institute of Siddha, Chennai, IND; 2 Department of Kuzhanthai Maruthuvam, National Institute of Siddha, Chennai, IND; 3 Department of Sattam Sarntha Maruthuvamum Nanju Maruthuvamum, Santhigiri Siddha Medical College and Hospital, Trivandrum, IND; 4 Department of Roga Nidanam, Sri Sairam Ayurveda Medical College and Research Centre, Chennai, IND; 5 Department of Sattam Sarntha Maruthuvamum Nanju Maruthuvamum, National Institute of Siddha, Chennai, IND

**Keywords:** cornu cutaneum, cutaneous horn, human papillomavirus, pachaieruvai, siddha medicine

## Abstract

A cutaneous horn, also known as cornu cutaneum, is a rare and usually benign growth that forms on the skin, extending outward from the outermost layer known as the stratum corneum. These growths are most often seen in older adults, particularly men, and are commonly linked to prolonged sun exposure, which can increase the risk of underlying conditions like squamous cell carcinoma (SCC). Other factors, such as infections caused by the human papillomavirus (HPV), have also been associated with their development. Treatment for cutaneous horns typically involves surgical excision, which is effective but may lead to issues such as scarring or infection. Other methods, like cryotherapy, laser therapy, topical treatments, and electrodesiccation with curettage, are also available. However, these approaches come with potential downsides, including pain, blistering, changes in skin pigmentation, and high costs, which can discourage some patients from seeking care. An alternative option is Siddha medicine, a traditional form of healing that offers a more natural, affordable, and well-tolerated approach to treating cutaneous horns. A 51-year-old male presented with a conical projection measuring 1 cm in height, located on the palmar aspect of the proximal interphalangeal joint of his right second finger, which had been present for three years. Based on clinical examination, the lesion was diagnosed as a cutaneous horn. The patient underwent treatment with a Siddha formulation called Pachaieruvai, which was applied externally for five consecutive days. In the end, the treatment proved effective, successfully destroying the growth within just 10 days. No adverse reactions or recurrence were observed during subsequent follow-ups, underscoring the intervention's effectiveness and safety.

## Introduction

Cutaneous horns, commonly referred to as cornu cutaneum, are rather uncommon benign growths that often appear as conical or cylindrical projections made up of compact keratin resembling an animal horn [1]. Unlike animal horns, which usually have a bony core, cutaneous horns are made up entirely of cornified proliferative keratinocytes and do not contain any bone structure [2]. These lesions are most frequently observed in older adults, with most cases reported in individuals aged 50 to 89 years [3]. However, they can also occur in younger individuals, including children. For instance, a case of a cutaneous horn on the eyelid of a 4-year-old child has been documented, highlighting the rare but possible occurrence of these lesions in the pediatric population [4]. Similarly, a 9-year-old boy with generalized Discoid Lupus Erythematosus (DLE) presented with multiple cutaneous horns, further demonstrating that these lesions are not exclusive to older adults [5]. Men are more frequently affected, especially in sun-exposed regions like the face and scalp, but cases in women, including a giant cutaneous horn on the leg of a 24-year-old female, have also been reported [6,7]. The lesions are most often associated with benign conditions, such as seborrheic keratosis or verruca vulgaris but can also be linked to premalignant or malignant conditions like actinic keratosis, squamous cell carcinoma, and basal cell carcinoma [6,8]. Human papillomavirus (HPV) has been linked to the development of cutaneous horn lesions, particularly those arising from verruca vulgaris (common warts), a condition caused by certain HPV strains [9]. In rare instances, cutaneous horns can develop in non-sun-exposed areas, including the forearm and penis [8,10]. Histopathological examination is crucial to determine the nature of the underlying pathology, ensuring accurate diagnosis and appropriate treatment [11]. When it comes to treating lesions, surgical excision is often the go-to method. This involves removing the lesion along with some surrounding tissue to ensure that it's completely excised. This technique is especially important when there's a suspicion of malignancy [12,13]. However, it's worth noting that surgical excision can come with certain risks, including scarring, infection, and the possibility of recurrence if the margins aren't fully cleared [13]. For smaller lesions, there are alternative options like electrosurgical excision and cryotherapy. Electrosurgical excision is less invasive, but it doesn’t always guarantee that all tissue is removed. Cryotherapy can be effective too, but it may lead to side effects such as hypopigmentation or blistering [13,14]. Topical treatments, like imiquimod or 5-fluorouracil, can also be an option for smaller or more superficial lesions. However, these treatments usually require several applications and can cause skin irritation [15]. One alternative worth considering is Siddha medicine. This traditional healing practice offers a more natural, cost-effective, and generally well-tolerated way to treat cutaneous horns. Siddha medicine is an ancient and respected medical tradition that provides a well-rounded and holistic approach to treating cutaneous horns. This case report aims to thoroughly document and assess how effective Siddha medicine, specifically Pachaieruvai, can be in treating this condition.

## Case presentation

A 51-year-old male presented with a conical projection measuring 1 cm in height, located on the palmar aspect of the proximal interphalangeal joint of his right second finger, which had been present for three years. The patient had not sought any prior medical intervention for his condition. On clinical examination, the lesion had a hard, keratotic texture and a brown color. There was no associated pain, bleeding, or discharge. The patient experiences discomfort while working. The rest of the physical examination was unremarkable.

Diagnosis

General and systemic examinations were normal. Based on the clinical presentation, the lesion was diagnosed as a cutaneous horn.

Management

**Figure 1:**
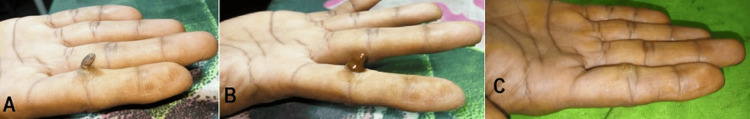
(A) Cutaneous horn before treatment, (B) during treatment, (C) and after treatment

The patient decided to remove the growth because it was causing ongoing irritation and significant discomfort. Before this decision, no treatment had been attempted. The patient opted for a traditional Siddha medicine called Pachaieruvai, which was applied topically for five consecutive days. During the treatment, the patient felt mild pain and a burning sensation at the application site, lasting about 30 minutes. These sensations were viewed as encouraging signs that the lesion was regressing. In the end, the treatment proved effective, successfully destroying the growth within just 10 days. The progress of cutaneous horn in the finger before (A), during (B), and after (C) treatment is shown in Figure 1.

Follow-up

A follow-up evaluation conducted 10 days after treatment confirmed the complete excision of the horn, with aesthetically pleasing results, and showed no signs of infection. When we checked in again at the three-month follow-up, everything looked good, there were no signs of adverse effects or any recurrence of the lesion.

## Discussion

This study takes an initial look at how to manage basal cell papilloma using Pachaieruvai, a traditional Siddha medicine, as outlined in ancient Siddha texts. The Pachaieruvai formulation consists of several components: vellaipadanam (arsenic trioxide), aridharam (arsenic trisulphide), thurusu (copper sulfate), karchunnam (calcium carbonate), and kungiliyam (resin of Shorea robusta) [16]. Arsenic compounds like arsenic trioxide and arsenic trisulfide have demonstrated significant antiproliferative effects on keratinocytes, the cells responsible for the excessive keratin production in cutaneous horns. These effects are primarily achieved through the induction of apoptosis, a programmed cell death process that helps reduce the abnormal cell proliferation associated with these lesions [17]. While copper sulfate isn't specifically mentioned in literature as a treatment for cutaneous horn, its therapeutic benefits for skin issues like umbilical granuloma hint at potential applications for cutaneous horn management. The effectiveness of copper sulfate in addressing skin lesions mainly comes from its caustic properties, which help in removing hyperkeratotic tissue [18]. *Karchunnam*, commonly known as calcium carbonate, is recognized for its cytotoxic properties [19].

Mode of action

The induction of apoptosis by the constituents of pachaieruvai may involve several cellular mechanisms, including DNA fragmentation, nuclear condensation, and the activation of caspase-3, a key enzyme in the apoptotic pathway. These processes culminate in a reduction of keratinocyte numbers, leading to the regression of the cutaneous horn. The ability of arsenic compounds to promote apoptosis and curb hyperkeratotic growth makes them a promising option for managing conditions like cutaneous horns.

Limitations of the study

Case studies focus on individual patients, which can limit our ability to generalize findings to larger populations. While they can highlight potential links between causes and outcomes, they don't establish definitive cause-and-effect relationships. The lack of controlled trials and large observational studies makes it difficult to identify specific factors that influence the progression of the cutaneous horn. Because of their limited scope, case studies may not capture the full range of manifestations or treatment responses associated with cutaneous horns. Additionally, they often spotlight rare or dramatic cases, which can introduce bias and skew our understanding of how frequently cutaneous horn occurs and how it progresses. Despite these limitations, case studies are still valuable for exploring treatment options, generating research hypotheses, and illustrating the complexities of managing cutaneous horn in real-world clinical settings. However, we should interpret their findings with caution and complement them with data from larger, more comprehensive studies to get a well-rounded view of cutaneous horns.

## Conclusions

This report examines the application of the Siddha methodology in treating cutaneous horns. The findings indicate that Siddha pharmacotherapy is highly effective in managing cutaneous horns. Integrating Pachaieruvai into this approach offers several benefits, including cost-effectiveness, time efficiency, reduced dosage requirements, and targeted localized therapeutic effects. Additionally, its economic feasibility renders it a compelling option within the therapeutic domain. Adopting a holistic healing strategy associated with traditional Siddha methods, which addresses the patient's issues and concerns, could be invaluable for treating cutaneous horn cases and ensuring the absence of recurrence. This case study presents a practical and economically viable approach to managing the everyday challenges of cutaneous horns, thereby enhancing the understanding of its treatment's medical and socioeconomic aspects. Furthermore, extensive clinical studies are imperative to gain deeper insights into these mechanisms. This represents a significant advancement in adopting a research-driven approach within a healthcare framework.
